# Immunization against recombinant GnRH-I alters ultrastructure of gonadotropin cell in an experimental boar model

**DOI:** 10.1186/1477-7827-11-63

**Published:** 2013-07-15

**Authors:** Fugui Fang, Shiping Su, Ya Liu, Yunhai Zhang, Yong Pu, Xijie Zhao, Yunsheng Li, Hongguo Cao, Juhua Wang, Jie Zhou, Xiaorong Zhang

**Affiliations:** 1 Anhui Provincial Laboratory of Animal Genetic Resources Protection and Breeding, College of Animal Sciences and Technology, Anhui Agricultural University, No. 130 of Changjiang West Road, Hefei, Anhui 230036, China; 2 Anhui Provincial Laboratory for Local Livestock and Poultry Genetic Resource Conservation and Bio-Breeding, No. 130 of Changjiang West Road, Hefei, Anhui 230036, China; 3 Engineering Research Center of Reproduction and breeding in Sheep of Anhui Province, No. 130 of Changjiang West Road, Hefei, Anhui 230036, China

**Keywords:** Ultrastructure, Gonadotropin, Boar, Immunization, GnRH-I

## Abstract

**Background:**

Gonadotropin cell is the main responsible for the secretion of follicle stimulating hormone (FSH) and luteinizing hormone (LH), and immunocastration reduces the concentrations of serum FSH and LH. A few studies have reported the histological structure of gonadotropin cells obtained from immunocastration animals at the light microscopy level. However, the ultrastructure of gonadotropin cells remains largely unexplored. The aim of this study was to evaluate and to compare ultrastructure of gonadotropin cell in gonadally intact boars and immunologically castrated male animals.

**Findings:**

In this study, serum and adenohypophysis tissue were collected from nine gonadally intact boars and nine male pigs treated with recombinant gonadotropin releasing hormone I (GnRH-I). Anti-GnRH-I antibodies in serum and the ultrastructure of gonadotropin cell in adenohypophysis were determined by enzymelinked immunosorbent assay and electron microscopy, respectively. The results demonstrated that active immunization against recombinant GnRH-I increased serum GnRH-I antibody levels (*P*<0.05). Ultramicroscopic analysis of gonadotropin cell revealed a decrease (*P*<0.05) in the number and size of the large granules and small granules in the recombinant GnRH-I immunized animals.

**Conclusions:**

We conclude that immunization against recombinant GnRH-I induces severe atrophy of granules in gonadotropin cell of boars, possibly reflecting GnRH-I regulation of gonadotropin cell.

## Findings

One promising alternative to surgical castration as a method of controlling undesirable behavior and aggression that has been researched for many years [1-3] is active immunization against (GnRH-I). Immunological castration uses the animal’s own immune system to suppress GnRH-I and thus shut down the stimulus to the testes resulting in an inhibition of testicular function. Many researchers report that immunization against GnRH-I significantly reduce serum concentrations of FSH and LH [4,5]. In sheep immunized against GnRH-I at prepubertal or peripubertal age, plasma LH concentrations were not restored after GnRH-I injection at a time when anti-GnRH-I antibodies are low [5,6] or not detectable [7]. GnRH-I or eCG treatment fails to reproductive function in GnRH-I immunized ewes [8]. Therefore, these findings have led to the suggestion that active immunization against GnRH-I disrupt the secretion of the gonadotropin cell in pituitary. Previous studies on pituitary obtained from immunocastration animals at the light microscopy level [8,9]. Nevertheless, to the best of our knowledge, there is no report to date on ultrastructural pituitary changes in the GnRH-I immunized animals. Thus, it is of interest to investigate the ultrastructure of gonadotropin cells in adenohypophysis of immunocastrated male pigs.

Fang et al. [10] used recombinant DNA technology to form maltose binding protein–gonadotropin releasing hormone I (MBP–GnRH-I6) vaccines, which had success in affecting the reproductive systems of pigs [11]. The objective of the present study was to evaluate the ultrastructure of gonadotrophin cells in MBP–GnRH-I6 immunized pigs.

## Methods

### Animals

Eighteen Chinese boars, reared at the DaDun Animal Farm, Shucheng, China, were used in the study. The study has been approved by Animal Care and Use Committee of Anhui Agricultural University. The animals were assigned randomly to two groups of the following treatments: MBP–GnRH-I6 immunization (n = 9) and MBP immunization (n = 9). All boars had access to food and water *ad libitum*.

### Preparation of antigens and immunization

MBP-GnRH-I6 was prepared and using recombinant DNA techniques as has been previously described [10]. Nine milligrams of MBP-GnRH-I6 or MBP was dissolved in 9 mL phosphate-buffered saline (PBS) and 9 mL of Al(OH)_3_ adjuvant (Tianbang, Nanjing, China). The first immunization was administered at 9 weeks of age by intramuscular injection of 2 mL of emulsion. The booster injection was given by the same route and at the same dose 8 weeks later. The pigs were slaughtered 8 weeks after the booster immunization.

### Analysis of anti-GnRH-I antibody

Blood samples were taken via the jugular vein when 9, 13, 17, 21 and 25 weeks old and centrifuged at 200 × *g* for 15 minutes at 4°C. Serum was harvested and stored at −80°C until assayed. The amount of anti-GnRH-I antibody in the collected serum from animals was measured as described by Fang et al. [11].

### Transmission electron microscopy of thin sections

After slaughter, the tissue of adenohypophysis was fixed in 2.5% glutaraldehyde for 4 to 6 hours, and post-fixation was accomplished in 1% osmium tetroxide for 1 hour. The samples were subjected to an alcohol dehydration series (30% 15 minutes, 50% 15 minutes, 70% 6–12 hours, 80% 15 minutes, 95% 15 minutes, 100% 40 minutes). The tissues were immersed in 1, 2-epoxypropane (Lingfeng Chemical Co. Ltd) for 30 minutes, and then transferred to 1, 2-epoxypropane and resin Epon812 (1:1) for 2 hours. Samples were individually embedded in Epon812 (Serva) for 2 hours. Resin blocks were solidified at 45°C for 12 hours and 65°C for at least 48 hours. Ultrathin sections (70 nm thick) were prepared from each tissue with an ultrathin section machine (LKBNUBA, NOVA) and blade (LKB2178, knife maker II, BROMMA). Sections were stained with 1% (w/v) methanolic uranyl acetate (Lanzhou State-owned Factory 404) for 30 minutes, and then washed three times in deionized water for a total of 15 minutes, and stained with lead citrate for 30 minutes.

The sections were rinsed in a stream of distilled water and dried prior to examination. Sections were visualized on a transmission electron microscopy (JEM-1230, Japan).

### Acquisition and analysis of data

Eight to 10 random sections were taken to represent tissue. The diameter and the number of the granules were measured using the specific software (Image-Pro plus 6.0). The data is expressed as mean ± standard deviation (SD). Statistical analysis was performed by the Student’s t-test. Significance was given at *P*<0.05.

## Results

**Figure 1:**
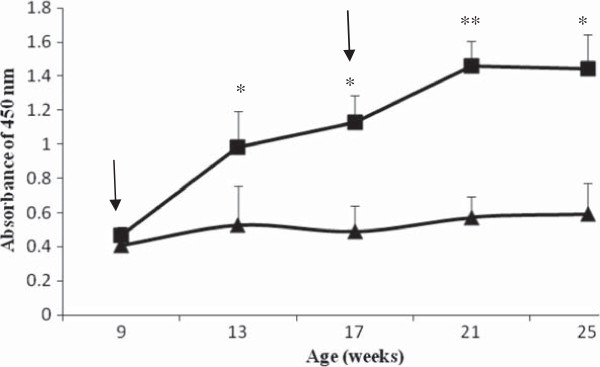
**High anti**-**GnRH**-**I antibody concentrations in boars immunized with MBP**–**GnRH**-**I6.** Microwell plates were coated with recombinant GnRH-I and incubated with sera collected from boars immunized with MBP–GnRH-I6 (n = 9) and MBP (n = 9). The results (mean ± SD) shown are the absorbance at 450nm for 1:500 dilution of sera. Arrows indicate time of immunizations. One star and two stars show *P* < 0.05 and *P* < 0.01, respectively. Black square and black triangle indicate MBP-GnRH-I6 immunized animals and MBP mock-immunized boars, respectively.

Results showed that serum level of the antibody against MBP–GnRH-I6 in vaccinated animals was increased significantly as compared with MBP mock-immunized boars (*P*<0.05) (Figure 1), suggesting MBP–GnRH-I6 immunization induced a strong anti-GnRH immune response.

**Figure 2:**
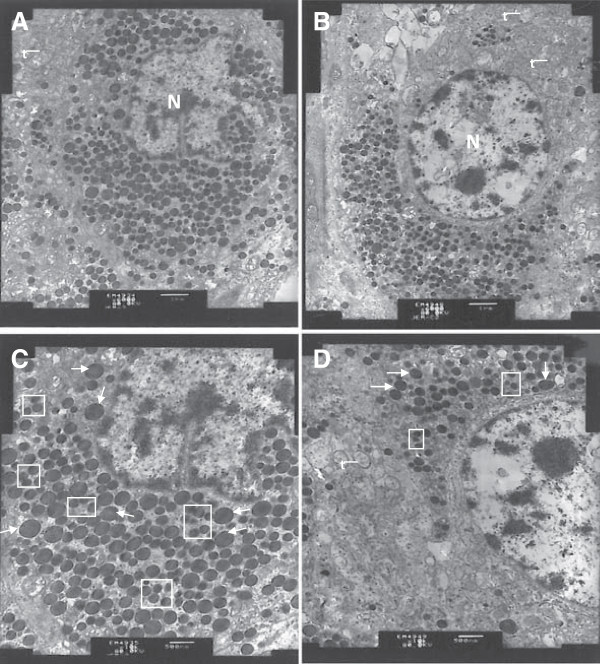
**Ultrastructure of the adenohypophysis showing gonadotropin cells in boars immunized with MBP**–**GnRH**-**I6** (**n** = **9**) **and MBP** (**n** = **9**). **A** and **C**: MBP mock-immunized boars; **B** and **D**: MBP-GnRH-I6 immunized male pigs. Cell organs are shown as the large granules (arrows), small granules (square area), mitochondrion (t) and nucleus (N). **A** and **B**: × 6000, scale bar = 1 μm; C and D: × 10000, scale bar = 500 nm.

A lot of large granules and small granules were found in cytoplasm of gonadotropin cell of adenohypophysis from electron microscopic images (Figure 2). The granules, round or oval in shape, main closely distributed at one side of nucleus (Figure 2). The gonadotropin cells showed evidence of severe changes in the granules. That is, the mean number and diameter of large granules and small granules in MBP mock- immunized boars (Figure 2A, C) were significantly more (*P*<0.05) than those of in MBP-GnRH-I6 immunized animals (Figure 2B, D) (Table 1).

**Table 1:** **The number and diameter of granules of gonadotropin cells in boars immunized with MBP**–**GnRH**-**I6**

**Group**	**Diameter of granules (nm)**		**The number of granules**	
	**Large**	**Small**	**Large**	**Small**
*MBP-GnRH-I6*	259.27±31.75^A^	157.75±19.45 ^A^	26.72±4.08 ^A^	152.56±9.78 ^A^
*MBP*	416.29±32.12^B^	216.89±17.19^B^	68.89±7.05^B^	201.05±12.89^B^

All data were shown as mean ± SD. Different superscripted letters in the column indicate a significant difference (*P*<0.05).

In conclusion, the results of the present study demonstrate that administration of recombinant GnRH-I to boars elicits clear increase in serum antibody levels and decrease in the number and diameter of the large granules and small granules in the gonadotropin cell.

## Abbreviations

FSH: Follicle stimulating hormone; LH: Luteinizing hormone; MBP–GnRH-I6: Maltose binding protein–gonadotropin releasing hormone I; MBP: Maltose binding protein; GnRH-I: Recombinant gonadotropin releasing hormone I.

## Competing interests

The authors declare that they have no competing interests.

## Authors’ contributions

FGF conceived of the study, and participated in its design and coordination and drafted the manuscript. SPS carried out the same work with FGF. YL participated in the design of the study and performed the statistical analysis. YHZ have been involved in drafting the manuscript. YP participated in the observation of electron microscopy. XJZ helped to draft the manuscript and critical revision of the manuscript. YSL have been revised the manuscript critically for important intellectual content. HGC carried out the analysis of data.. JHW participated in the immunization of animals and the determination of antibody. JZ participated in analysis of electron microscopic images and the design and drafted the manuscript. XRZ participated in the design and coordination and helped to draft the manuscript and provided the fund. All authors read and approved the final manuscript.
